# Recommendations for Processing Head CT Data

**DOI:** 10.3389/fninf.2019.00061

**Published:** 2019-09-04

**Authors:** John Muschelli

**Affiliations:** Department of Biostatistics, Johns Hopkins Bloomberg School of Public Health, Baltimore, MD, United States

**Keywords:** CT, image processing, image normalization, image analysis, non-contrast CT, image de-identification, neuroimaging, neuroimaging analysis

## Abstract

Many research applications of neuroimaging use magnetic resonance imaging (MRI). As such, recommendations for image analysis and standardized imaging pipelines exist. Clinical imaging, however, relies heavily on X-ray computed tomography (CT) scans for diagnosis and prognosis. Currently, there is only one image processing pipeline for head CT, which focuses mainly on head CT data with lesions. We present tools and a complete pipeline for processing CT data, focusing on open-source solutions, that focus on head CT but are applicable to most CT analyses. We describe going from raw DICOM data to a spatially normalized brain within CT presenting a full example with code. Overall, we recommend anonymizing data with Clinical Trials Processor, converting DICOM data to NIfTI using dcm2niix, using BET for brain extraction, and registration using a publicly-available CT template for analysis.

## 1. Introduction

Many research applications of neuroimaging use magnetic resonance imaging (MRI). MRI allows researchers to study a multitude of applications and diseases, including studying healthy volunteers. Clinical imaging, however, relies heavily on X-ray computed tomography (CT) scans for diagnosis and prognosis. Studies using CT scans cannot generally recruit healthy volunteers or large non-clinical populations due to the radiation exposure and lack of substantial benefit. As such, much of head CT data is gathered from prospective clinical trials or retrospective studies based on health medical record data and hospital picture archiving and communication system (PACS). We discuss transforming this data from clinical to research data and provide some recommendations and guidelines from our experience with CT data similar insights from working with MRI studies. We will discuss existing software options, focusing on open-source tools, for neuroimaging in general and those that are specific to CT throughout the paper.

We will focus on aspects of quantitatively analyzing the CT data and getting the data into a format familiar to most MRI neuroimaging researchers. Therefore, we will not go into detail of imaging suites designed for radiologists, which may be proprietary and quite costly. Moreover, we will be focusing specifically on non-contrast head CT data, though many of the recommendations and software is applicable to images of other areas of the body.

The pipeline presented here is similar to that of Dhar et al. (2018). We aim to discuss the merits of each part of the pipeline with a set of choices that have available code. In addition, we present a supplement with a working example, including code, to go from DICOM data to a spatially-normalized brain image. We also touch on points relevant to de-identification of the data, not only from DICOM metadata, but also removing identifiable information from the image itself such as the face. Overall, we aim to discuss the suite of tools available, many of which built specifically for MRI, but provide slight modifications if necessary to have these work for head CT.

## 2. Data Organization

Most of the data coming from a PACS is in DICOM (Digital Imaging and Communications in Medicine) format. Generally, DICOM files are a combination of metadata (i.e., a header) about an image and the individual pixel data, many times embedded in a JPEG format. The header has a collection of information, usually referred to as fields or tags. Tags are usually defined by a set of 2 hexadecimal numbers, which are embedded as 4 alphanumeric characters. For example, (0008,103E) denotes the SeriesDescription tag for a DICOM file. Most DICOM readers extract and use these tags for filtering and organizing the files. The pixel data is usually given in the axial orientation in a high resolution (e.g., 0.5 mm^2^) grid of 512 x 512 pixels.

We will use the phrase scanning session (as opposed to “study” and reserve study to denote a trial or analysis), a series for an individual scan, and a slice for an individual picture of the brain. Each series (Series Instance UID tag) and scanning session (Study Instance UID tag) should have a unique value in the DICOM header that allows DICOM readers to organize the data by scanning session and series. The following sections will discuss data organization and data formats.

### 2.1. DICOM Anonymization

One of the common issues with DICOM data is that a large amount of protected health information (PHI) can be contained in the header. DICOM is a standard where individual fields in the header contain the same values across different scanners and sites, but only if that manufacturer and site are diligent to ascribing to the DICOM standard. Though many DICOM header fields are consistent across neuroimaging studies, a collection of fields may be required to obtain the full amount of data required for analysis. Moreover, different scanning manufacturers can embed information in non-standard fields. The goal is to remove these fields if they contain PHI, but retain these fields if they embed relevant information of the scan for analysis. These fields then represent a challenge to anonymization without loss of crucial information if the data do not conform to a standard across scanning sites, manufacturers, or protocols.

We will discuss reading in DICOM data and DICOM header fields in the next section. Reading DICOM data may be necessary for extracting information, but many times the data must be transferred before analysis. Depending on the parties receiving the data, anonymization of the data must be done first. Aryanto et al. (2015) provides a look at a multitude of options for DICOM anonymization and recommend the RSNA MIRC Clinical Trials Processor (CTP, https://www.rsna.org/research/imaging-research-tools) a, cross-platform Java software, as well as the DICOM library (https://www.dicomlibrary.com/) upload service. We also recommend the DicomCleaner cross-platform Java program as it has similar functionality. Bespoke solutions can be generated using dcm4che (such as dcm4che-deident, https://www.dcm4che.org/) and other DICOM reading tools (discussed below), but many of these tools have built-in capabilities that are difficult to add (such as removing PHI embedded in the pixel data, aka “burned in”).

#### 2.1.1. A Note on De-identification: Time Between Scans

Although most of the presented solutions are good at anonymization and de-identification of the header information, only a few such as CTP, have the utilities required for longitudinal preservation of date differences. Dates are considered removable identifiable information under HIPAA, some clinical trials and other studies rely on serial CT imaging data, and the differences between times are crucial to determine when events occur or are used in analysis.

### 2.2. Publicly Available Data

With the issues of PHI above coupled with the fact that most CT data is acquired clinically and not in a research setting, there is a dearth of publicly available data for head CT compared to head MRI. Sites for radiological training such as Radiopedia (https://radiopaedia.org/) have many cases of head CT data, but these are converted from DICOM to standard image formats (e.g., JPEG) so crucial information, such as Hounsfield Units and pixel dimensions, are lost.

Large repositories of head CT data do exist, though, and many in DICOM format, with varying licenses and uses. The CQ500 (Chilamkurthy et al., 2018) dataset provides approximately 500 head CT scans with different clinical pathologies and diagnoses, with a non-commercial license. All examples in this article use data from 2 subjects within the CQ500 data set. The Cancer Imaging Archive (TCIA) has hundreds of CT scans, many cases with brain cancer. TCIA also has a RESTful (representational state transfer) interface, which allows cases to be downloaded in a programmatic way; for example, the TCIApathfinder R package (Russell, 2018) and Python tciaclient module provide an interface. The Stroke Imaging Repository Consortium (http://stir.dellmed.utexas.edu/) also has head CT data available for stroke. The National Biomedical Imaging Archive (NBIA, https://imaging.nci.nih.gov) demo provides some head CT data, but are mostly duplicated from TCIA. The NeuroImaging Tools & Resources Collaboratory (NITRC, https://www.nitrc.org/) provides links to many data sets and tools, but no head CT images at this time. The RIRE (Retrospective Image Registration Evaluation, http://www.insight-journal.org/rire/) and MIDAS (http://www.insight-journal.org/midas) projects have small set of publicly available head CT (under 10 participants).

#### 2.2.1. Reading DICOM Data

Though MATLAB has an extensive general imaging suite, including SPM (Penny et al., 2011), we will focus on R (R Core Team, 2018) Python (Python Software Foundation, https://www.python.org/), and other standalone software. The main reasons are that R and Python are free, open source, and have a lot of functionality with neuroimaging and interface with popular imaging suites. We are also lead the Neuroconductor project (https://neuroconductor.org/) (Muschelli et al., 2018), which is a repository of R packages for medical image analysis. Other imaging platforms such as the Insight Segmentation and Registration Toolkit (ITK) are well-maintained, useful pieces of software that can perform many of the operations that we will be discussing. We will touch on some of this software with varying levels. We aim to present software that we have had used directly for analysis or preprocessing. Also, other papers and tutorials discuss the use of these tools in analysis (https://neuroconductor.org/tutorials).

For reading DICOM data, there are multiple options. The oro.dicom (Whitcher et al., 2011) and radtools (Russell and Ghosh, 2019) R packages, pydicom Python module (Mason, 2011), MATLAB imaging toolbox, and ITK (Schroeder et al., 2003) interfaces can read DICOM data amongst others. The DICOM toolkit dcmtk (Eichelberg et al., 2004) has multiple DICOM manipulation tools, including dcmconv to convert DICOM files to other imaging formats. Though most imaging analysis tools can read in DICOM data, there are downsides to using the DICOM format. In most cases, a DICOM file is a single slice of the full 3D image series. This separation can be cumbersome on data organization if using folder structures. As noted above, these files also can contain a large amount of PHI. Some image data may be compressed, such as JPEG2000 format. Alternatively, if data are not compressed, file storage is inefficient. Most importantly, many imaging analyses perform 3-dimensional (3D) operations, such as smoothing. Thus, putting the data into a different format that handles 3D images as 1 compressed file is desirable. We present examples of reading DICOM data above, but generally recommend using 3D imaging formats and using the above tools to read the DICOM header information.

### 2.3. Converting DICOM to NIfTI

Many different general 3D medical imaging formats exist, such as ANALYZE, NIfTI, NRRD, and MNC. We recommend the NIfTI format, as it can be read by nearly all medical imaging platforms, has been widely used, has a format standard, can be stored in a compressed format, and is how much of the data is released online. Moreover, we will present specific software to convert DICOM data and the recommended software (dcm2niix) outputs data in a NIfTI file.

Many sufficient and complete solutions exist for DICOM to NIfTI conversion. Examples include dicom2nifti in the oro.dicomR package, pydicom, dicom2nifti in MATLAB, and using large imaging suites such as using ITK image reading functions for DICOM files and then write NIfTI outputs. We recommend dcm2niix (https://github.com/rordenlab/dcm2niix) (Li et al., 2016) for CT data for the following reasons: (1) it works with all major scanners, (2) incorporates gantry-tilt correction for CT data, (3) can handle variable slice thickness, (4) is open-source, (5) is fast, (6) is an actively maintained project, and (7) works on all 3 major operating systems (Linux/OSX/Windows). Moreover, the popular AFNI neuroimaging suite includes a dcm2niix program with its distribution. Interfaces exist, such as the dcm2niir (Muschelli, 2018) package in R and nipypePython module (Gorgolewski et al., 2011). Moreover, the divest package (Clayden and Rorden, 2018) wraps the underlying code for dcm2niix to provide the same functionality of dcm2niix, along with the ability to manipulate the data for more versatility.

**Figure 1:**
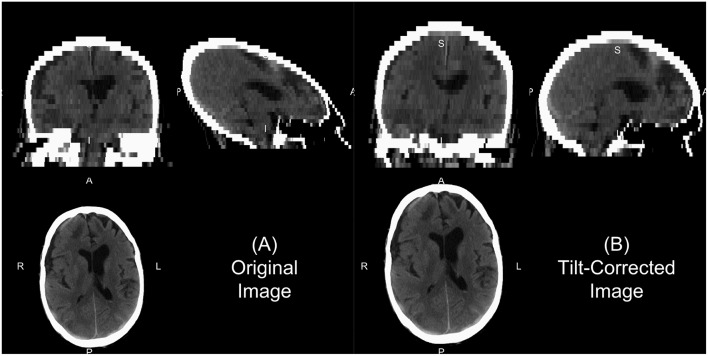
Example of gantry-tilt correction. Using “dcm2niix,” we converted the DICOM files to a NIfTI file, which had a 30 degree tilt. The output provides the uncorrected image **(A)** and the tilt-corrected image **(B)**. We see that the reconstructed image without correction appears fine within the axial plane, but out of plane has an odd 3D shape. This shape will be corrected with an affine transformation, which is done in the conversion, correcting the image as seen in **(B)**.

We will describe a few of the features of dcm2niix for CT. In some head CT scans, the gantry is tilted to reduce radiation exposure to non-brain areas, such as the eyes. Thus, the slices of the image are at an oblique angle. If slice-based analyses are done or an affine registration (as this tilting is a shearing) are applied to the 3D data, this tilting may implicitly be corrected. This tilting causes issues for 3D operations as the distance of the voxels between slices is not correct and especially can show odd visualizations (Figure 1A). The dcm2niix output returns both the corrected and non-corrected image (Figure 1). As the correction moves the slices to a different area, dcm2niix may pad the image so that the entire head is still inside the field of view. As such, this may cause issues with algorithms that require the 512 x 512 axial slice dimensions. Though less common, variable slice thickness can occur in reconstructions where only a specific area of the head is of interest. For example, an image may have 5 mm slice thicknesses throughout the image, except for areas near the third ventricle, where slices are 2.5 mm thick. To correct for this, dcm2niix interpolates between slices to ensure each image has a consistent voxel size. Again, dcm2niix returns both the corrected and non-corrected image.

Once converted to NIfTI format, one should ensure the scale of the data. Most CT data is between −1024 and 3071 Hounsfield Units (HU). Values less than −1024 HU are commonly found due to areas of the image outside the field of view that were not actually imaged. One first processing step would be to Winsorize the data to the [−1024, 3071] range. After this step, the header elements scl_slope and scl_inter elements of the NIfTI image should be set to 1 and 0, respectively, to ensure no data rescaling is done in other software. Though HU is the standard format used in CT analysis, negative HU values may cause issues with standard imaging pipelines built for MRI, which typically have positive values. Rorden (CITE) proposed a lossless transformation, called Cormack units, which have a minimum value of 0. The goal of the transformation is to increase the range of the data that is usually of interest, from −100 to 100 HU and is implemented in the Clinical toolbox (discussed below). Most analyses are done using HU, however.

### 2.4. Convolution Kernel

**Figure 2:**
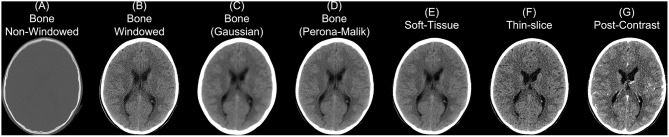
Different series for a scanning study. Here we present different non-contrast head CT exported from a PACS. We display a reconstructed scan with a bone convolution kernel **(A)**, showing bright areas of the skull, which can be investigated for breaks or fractures. When applying a window of 0–100 Hounsfield units (HU) of this image, we see the image resolution **(B)**. Using a Gaussian **(C)** or Perona-Malik **(D)** smoother, we see the resulting image smoothness is similar to the image reconstructed with a soft-tissue convolution kernel **(E)**. Images **(A–E)** had a slice thickness of 5 mm. The thin-slice scan **(F)** had a slice thickness of 0.62 mm and a soft-tissue convolution kernel. CT scan with contrast agent **(G)** to show how the contrast affects the CT image.

Though we discuss CT as having more standardized Hounsfield unit values, this does not imply CT scans cannot have vastly different properties depending on parameters of scanning and reconstruction. One notable parameter in image reconstruction is the convolution kernel [i.e., filter, DICOM field (0018,1210)] used for reconstruction. We present slices from an individual subject from the CQ500 (Chilamkurthy et al., 2018) dataset in Figure 2. Information on which kernel was used, and other reconstruction parameter information can be found in the DICOM header. The kernel is described usually by the letter “H” (for head kernel), a number indicating image sharpness (e.g., the higher the number, the sharper the image, the lower the number, the smoother the image), and an ending of “s” (standard), “f” (fast), “h” for high resolution modes (Siemens SOMATOM Definition Application Guide), though some protocols simply name them “soft-tissue,” “standard,” “bone,” “head,” or “blood,” amongst others. The image contrast can depend highly on the kernel, and “medium smooth” kernels (e.g., H30f, H30s) can provide good contrast in brain tissue (Figure 2E). Others, such as “medium” kernels (e.g., H60f, H60s) provide contrast in high values of the image, such as detecting bone fractures (Figure 2A), but not as good contrast in brain tissue (Figure 2B). Thus, when combining data from multiple sources, the convolution kernel may be used to filter, stratify, or exclude data.

Moreover, the noise and image contrast can be different depending on the image resolution of the reconstruction. Most standard head CT scans have high resolution within the axial plane (e.g., 0.5 x 0.5 mm). Image reconstructions can have resolution in the interior-superior plane (e.g., slice thickness) anywhere from 0.5mm (aka “thin-slice,” Figure 2F) to 2.5 mm, to 5 mm, where 5 mm is fairly common. The larger the slice thicknesses are, the smoother the reconstruction (as areas are averaged). Also, the added benefit for radiologists and clinicians are that fewer slices are needed to be reviewed for pathology or to get a comprehensive view of the head. In research, however, these thin-slice scans can get better estimates of volumes of pathology, such as a hemorrhage (CITE), or other brain regions. Moreover, when performing operations across images, algorithms may need to take this differing resolution, and therefore image dimensions, into account. We will discuss image registration in the data preprocessing as one way to harmonize the data dimensions, but registration does not change the inherent smoothness or resolution of the original data.

In some instances, only certain images are available for certain subjects. For example, most of the subjects have a non-contrast head CT with a soft-tissue convolution kernel, whereas some only have a bone convolution kernel. Post-processing smoothing can be done, such as 3D Gaussian (Figure 2C) or anisotropic (Perona-Malik) smoothing (Perona and Malik, 1990; Figure 2D). This process changes the smoothness of the data, contrast of certain areas, can cause artifacts in segmentation, but can make the within-plane properties similar for scans with bone convolution kernel reconstructions compared to soft-tissue kernels in areas of the brain (Figure 2E).

### 2.5. Contrast Agent

Though we are discussing non-contrast scans, head CT scans with contrast agent are common. The contrast/bolus agent again should be identified in the DICOM header field (0018,0010), but may be omitted. The contrast changes CT images, especially where agent is delivered, notably the vascular system of the brain (Figure 2G). These changes may affect the steps recommended in the next section of data preprocessing, where thresholds may need to be adjusted to include areas with contrast which can have higher values than the rest of the tissue (e.g., > 100 HU; Figure 2G).

## 3. Data Preprocessing

Now that the data is in a standard file format, we can discuss data preprocessing. As the data are in NIfTI format, most software built for MRI and other imaging modalities should work, but adaptations and other considerations may be necessary.

### 3.1. Bias-Field/Inhomogeneity Correction

In MRI, the scan may be contaminated by a bias field or set of inhomogeneities. This field is generally due to inhomogeneities/inconsistencies in the MRI coils or can be generated by non-uniform physical effects on the coils, such as heating. One of the most common processing steps done first is to remove this bias field. In many cases, these differences can more general be considered non-uniformities, in the sense that the same area with the same physical composition and behavior may take on a different value if it were in a different spatial location of the image. Though CT data has no coil or assumed bias field due to the nature of the data, one can test if trying to harmonize the data spatially with one of these correction procedures improves performance of a method. Though we do not recommend this procedure generally, as it may reduce contrasts between areas of interest, such as hemorrhages in the brain, but has been used to improve segmentation (Cauley et al., 2018). We would like to discuss potential methods and CT-specific issues.

Overall, the assumptions of this bias field are that it is multiplicative and is smoothly varying. One of the most popular inhomogeneity corrections are the non-parametric non-uniformity normalization (e.g., N3; Sled et al., 1998) and its updated improvement N4 (Tustison et al., 2010) in ANTs, though other methods exist in FSL (Zhang et al., 2001) and other software (Ashburner and Friston, 1998; Belaroussi et al., 2006). Given the assumption of the multiplicative nature of the field, N4 performs an expectation–maximization (EM) algorithm on the log-transformed image, assuming a noise-free system. As CT data in HU has negative values, the log transform is inappropriate. Pre-transforming or shifting the data values may be necessary to perform this algorithm, though these transforms may affect performance. Moreover, artifacts or objects (described below), such as the bed, may largely effect the estimation of the field and segmentation may be appropriate before running these corrections, such as brain extraction or extracting only subject-related data and not imaged hardware. The ANTsR package (https://github.com/ANTsX/ANTsR) provides the n4BiasFieldCorrection function in R; ANTsPy (https://github.com/ANTsX/ANTsPy) and NiPype (Gorgolewski et al., 2011) provide n4_bias_field_correction and N4BiasFieldCorrection in Python, respectively.

### 3.2. Brain Extraction in CT

**Figure 3:**
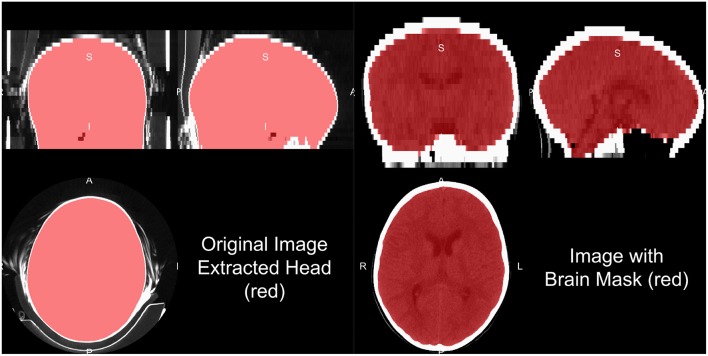
Human and brain extraction results. Here we present a 5 mm slice, non-contrast head CT with a soft-tissue convolution kernel. The left figure represents the CT image, showing all the areas imaged, overlaid with the extracted head mask as described in the section of “Brain Extraction in CT.” The right hand side is the image overlaid with a brain mask. The brain mask was created using an adaptation of the Brain Extraction Tool (BET) from FSL, published by Muschelli et al. (2015).

Head CT data typically contains the subject's head, face, and maybe neck and other lower structures, depending on the field of view. Additionally, other artifacts are typically present, such as the pillow the subject's head was on, the bed/gurney, and any instruments in the field of view. We do not provide a general framework to extract the complete head from hardware, but provide some recommendations for working heuristics. Typically the range of data for the brain and facial tissues are within −100 to 300 HU, excluding the skull, other bones, and calcificiations. Creating a mask from values from the −100 to 1000 HU range tends to remove some instruments, the pillow, and the background. Retaining the largest connected component will remove high values such as the bed/gurney, filling holes (to include the skull), and masking the original data with this resulting mask will return the subject (Figure 3).

Note, care must be taken whenever a masking procedure is used as one standard way is to set values outside an area of interest to 0. With CT data 0 HU is a real value of interest: if all values are set to 0 outside the mask, the value of 0 is aliased to both 0 HU and outside of mask. Either transforming the data into Cormack units, adding a value to the data (such as 1025) then setting values to 0, or using NaN are recommended in values not of interest.

One of the most common steps in processing imaging of the brain is to remove non-brain structures from the image. Many papers present brain extracted CT images, but do not always disclose the method of extraction. We have published a method that uses the brain extraction tool (BET) from FSL, originally built for MRI, to perform brain extraction (Muschelli et al., 2015) with the CT_Skull_Strip function in the ichsegR package (Muschelli, 2019). An example of this algorithm performance on a 5 mm slice, non-contrast head CT with a soft-tissue convolution kernel is seen in Figure 3, which extracts the relevant areas for analysis. Recently, convolutional neural networks and shape propagation techniques have been quite successful in this task (Akkus et al., 2018) and models have been released (https://github.com/aqqush/CT_BET). Overall, much research can still be done in this area as traumatic brain injury (TBI) and surgery, such as craniotomies or craniectomies, can cause these methods to potentially fail. Overall, however, large contrast between the skull and brain tissue and standardized Hounsfield Units can make brain segmentation an easier task than in MRI.

#### 3.2.1. Tissue-Class Segmentation

In many structural MRI applications, the next step may be tissue-class segmentation, denoting areas of the cerebrospinal fluid (CSF), white matter and gray matter. Though Cauley et al. (2018) provides an example of tissue-class segmentation of CT scans using available software (intended for MRI) (Zhang et al., 2001), we will not cover them in detail here. One potential issue is the contrast between white and gray matter is much lower than compared to MRI T1-weighted imaging. Rather than tissue-class segmentation, a number of examples exist of determining CSF space from CT, including scans with pathology (Hacker and Artmann, 1978; Liu et al., 2010; Li et al., 2012; Poh et al., 2012; Ferdian et al., 2017; Patel et al., 2017; Dhar et al., 2018). These methods sometimes segment the CSF from the brain, including areas of the subarachnoid space, only the ventricles, or some combination of the two. Moreover, these CT-specific methods have not released open-source implementations or trained models for broad use.

### 3.3. Removal of Identifiable Biometric Markers: Defacing

As part of the Health Insurance Portability and Accountability Act (HIPAA) in the United States, under the “Safe Harbor” method, releasing of data requires the removal a number of protected health information (PHI) (Centers for Medicare & Medicaid Services, 1996). For head CT images, a notable identifier is “Full-face photographs and any comparable images”. Head CT images have the potential for 3D reconstructions, which likely fall under this PHI category, and present an issue for reidentification of participants (Schimke and Hale, 2015). Thus, removing areas of the face, called defacing, may be necessary for releasing data. If parts of the face and nasal cavities are the target of the imaging, then defacing may be an issue. As ears may be a future identifying biometric marker, and dental records may be used for identification, these areas may desirable to remove (Cadavid et al., 2009; Mosher, 2010).

The obvious method for image defacing is to perform brain extraction we described above. If we consider defacing to be removing parts the face, while preserving the rest of the image as much as possible, this solution is not sufficient. Additional options for defacing exist such as the MRI Deface software (https://www.nitrc.org/projects/mri_deface/), which is packaged in the FreeSurfer software and can be run using the mri_deface function from the freesurfer R package (Bischoff-Grethe et al., 2007; Fischl, 2012). We have found this method does not work well out of the box on head CT data, including when a large amount of the neck is imaged.

Registration methods involve registering images to the CT and applying the transformation of a mask of the removal areas (such as the face). Examples of this implementation in Python modules for defacing are pydeface (https://github.com/poldracklab/pydeface/tree/master/pydeface) and mridefacer (https://github.com/mih/mridefacer). These methods work since the registration from MRI to CT tends to performs adequately, usually with a cross-modality cost function such as mutual information. Other estimation methods such as the Quickshear Defacing method rely on finding the face by its relative placement compared to a modality-agnostic brain mask (Schimke and Hale, 2011). The fslr R package implements both the methods of pydeface and Quickshear. The ichseg R package also has a function ct_biometric_mask that tries to remove the face and ears based registration to a CT template (described below). Overall, removing potential biometric markers from imaging data should be considered when releasing data and a number of methods exist, but do not guarantee complete de-identification and may not work directly with CT without modification.

### 3.4. Registration to a CT Template

Though many analyses in clinical data may be subject-specific, population-level analyses are still of interest. Some analyses want spatial results at the population-level, which require registration to a population template. One issue with these approaches is that most templates and approaches rely on an MRI template. These templates were developed by taking MRI scans of volunteers, which again is likely unethical with CT due to the radiation exposure risk without other benefits. To create templates, retrospective searches through medical records can provide patients who came in with symptoms warranting a CT scan, such as a migraine, but had a diagnosis of no pathology or damage. Thus, these neuro-normal scans are similar to that of those collected those in MRI research studies, but with some important differences. As these are retrospective, inclusion criteria information may not be easily obtainable if not clinically collected, scanning protocols and parameters may vary, even within hospital and especially over time, and these patients still have neurological symptoms. Though these challenges exist, with a large enough patient population and a research consent at an institution, these scans can be used to create templates and atlases based on CT. To our knowledge, the first publicly available head CT template exists was released by Rorden et al. (2012), for the purpose of spatial normalization/registration.

**Figure 4:**
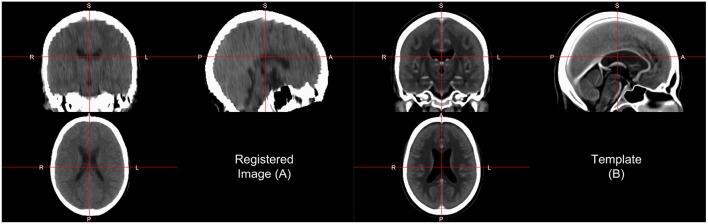
Image registration result. Here we displayed the scan **(A)** registered to a CT template **(B)** from Rorden et al. (2012). The registration by first doing an affine registration, followed by symmetric normalization (SyN), a non-linear registration implemented in ANTsR. The registration was done with the skull on the image and the template. We see areas of the image that align generally well, but may not be perfect.

One interesting aspect of CT image registration is again that CT data has units within the same range. To say they are uniformly standardized is a bit too strong as tomography and other confounds can impact units. Thus, it is our practice to think of them as more standardized than MRI. This standardization may warrant or allow the user different search and evaluation cost functions for registration, such as least squares. We have found that normalized mutual information (NMI) still performs well in CT-to-CT registration and should be at least considered when using CT-to-MRI or CT-to-PET registration. Along with the template above, Rorden et al. (2012) released the Clinical toolbox (https://github.com/neurolabusc/Clinical) for SPM to allow researchers to register head CT data to a standard space. However, as the data are in the NIfTI format, almost all image registration software should work, though one should consider transforming the units using Cormack units or other transformations as negative values may implicitly be excluded in some software built for MRI registration. We have found using diffeomorphic registrations such as symmetric normalization (SyN) from ANTs and ANTsR with NMI cost functions to perform well. We present results of registering the head CT presented in brain extraction to the template from Rorden et al. (2012) using SyN in Figure 4.

In some cases, population-level analyses can be done, but while keeping information at a subject-specific level. For example, registration from a template to a subject space can provide information about brain structures that can be aggregated across people. For example, one can perform a label fusion approach to CT data to infer the size of the hippocampus and then analyze hippocampi sizes across the population. Numerous label fusion approaches exist (Collins and Pruessner, 2010; Langerak et al., 2010; Sabuncu et al., 2010; Asman and Landman, 2013; Wang et al., 2013), but rely on multiple templates and publicly available segmented CT images are still lacking. Additionally, the spatial contrast in CT is much lower than T1-weighted MRI for image segmentation. Therefore, concurrent MRI can be useful. One large issue is that any data gathered with concurrent MRI the high variability in MRI protocol done if it is not generally standardized within or across institution. We see these limits as a large area of growth and opportunity in CT image analysis.

### 3.5. Pipeline

Overall, our recommended pipeline is as follows:

1. Use CTP or DicomCleaner to organize and anonymize the DICOM data from a PACS.
2. Extract relevant header information for each DICOM, using software such as dcmdump from dcmtk and store, excluding PHI.
3. Convert DICOM to NIfTI using dcm2niix, which can create brain imaging data structure (BIDS) formatted data (Gorgolewski et al., 2016). Use the tilt-corrected and data with uniform voxel size.

After, depending on the purpose of the analysis, you may do registration then brain extraction, brain extraction then registration, or not do registration at all. If you are doing analysis of the skull, you can also use brain extraction as a first step to identify areas to be removed. For brain extraction, run BET for CT or CT_BET (especially if you have GPUs for the neural network). If registration is performed, keeping the transformations back into the native, subject space is usually necessary as many radiologists and clinicians are comfortable to subject-specific predictions or segmentations. Converting the data from NIfTI back to DICOM is not commonly done, but is possible as most PACS are built for DICOM data.

## 4. Conclusions

We present a simple pipeline for preprocessing (see Data Sheet 1) of head CT data, along with software options of reading and transforming the data. We have found that many tools exist for MRI and are applicable to CT data. Noticeable differences exist between the data in large part due to the collection setting (research vs. clinical), data access, data organization, image intensity ranges, image contrast, and population-level data. As CT scans provide fast and clinically relevant information and with the increased interest in machine learning in medical imaging data, particularly deep learning using convolutional neural networks, research and quantitative analysis of head CT data is bound to increase. We believe this presents an overview of a useful set of tools and data for research in head CT.

For research using head CT scans to have the level of interest and success as MRI, additional publicly available data needs to be released. We saw the explosion of research in MRI, particularly functional MRI, as additional data were released and consortia created truly large-scale studies. This collaboration is possible at an individual institution, but requires scans to be released from a clinical population, where consent must be first obtained, and upholding patient privacy must be a top priority. Large internal data sets likely exist, but institutions need incentives to release these data sets to the public. Also, though institutions have large amounts of rich data, general methods, and applications require data from multiple institutions as parameters, protocols, and population characteristics can vary widely.

One of the large hurdles after creating automated analysis tools or supportive tools to help radiologists and clinicians is the reintegration of this information into the healthcare system. We do not present answers to this difficult issue, but note that these tools first need to be created to show cases where this reintegration can improve patient care, outcomes, and other performance metrics. We hope the tools and discussion we have provided advances those efforts for researchers starting in this area.

All of the code used to generate the figures in this paper is located at https://github.com/muschellij2/process_head_ct. The code uses packages from Neuroconductor in R. All data presented was from the CQ500 data set, which can be downloaded from http://headctstudy.qure.ai/dataset.

## Data Availability

Publicly available datasets were analyzed in this study. This data can be found here: http://headctstudy.qure.ai/dataset.

## Author Contributions

The author confirms being the sole contributor of this work and has approved it for publication.

### Conflict of Interest Statement

The author declares that the research was conducted in the absence of any commercial or financial relationships that could be construed as a potential conflict of interest.
