# Supplementary material for: Recommendations for Processing Head CT Data
Source: Front Neuroinform. 2019 Sep 4;13:61. doi: 10.3389/fninf.2019.00061 (PMC6738271; doi:10.3389/fninf.2019.00061)
Supplement: Supplementary file 1 [file Data_Sheet_1.pdf]

Skull Stripping and Registration of Head CT Data

Goal

In this tutorial, we will discuss skull-stripping (or brain-extracting) X-ray computed tomography (CT) scans. We will use data from TCIA (http://www.cancerimagingarchive.net/). The entire pipeline goes from raw DICOM data, converts it to NIfTI images, performs brain extraction, and then spatially normalizes the brain to a template using non-linear registration. All of the packages are open source and are available through CRAN or Neuroconductor (https://neuroconductor.org/) for the R programming language. We extract data from TCIA from the `TCIApathfinder` R package.

Installing Packages

In order to run all the code in this tutorial, these packages need to be installed. The following code should install all the packages.

```
install.packages(c("TCIApathfinder", "dplyr"))
source("https://neuroconductor.org/neurocLite.R")
neuro_install(c("dcm2niir", "ichseg", "fslr", "extrantsr"))
```

Using TCIApathfinder

In order to use `TCIApathfinder`, please see the vignette to obtain API keys (Russell et al. 2018). Here we will look at the collections of data available given the code below:

```
library(TCIApathfinder)
series_instance_uid = "1.3.6.1.4.1.14519.5.2.1.2857.3707.893926543922125108620513439908"
download_unzip_series = function(series_instance_uid,
                                 verbose = TRUE) {
  tdir = tempfile()
  dir.create(tdir, recursive = TRUE)
  tfile = tempfile(fileext = ".zip")
  tfile = basename(tfile)
  if (verbose) {
    message("Downloading Series")
  }
  res = save_image_series(
    series_instance_uid = series_instance_uid,
    out_dir = tdir,
    out_file_name = tfile)
  if (verbose) {
    message("Unzipping Series")
  }
  stopifnot(file.exists(res$out_file))
  tdir = tempfile()
  dir.create(tdir, recursive = TRUE)
  res = unzip(zipfile = res$out_file, exdir = tdir)
  L = list(files = res,
           dirs = unique(dirname(normalizePath(res))))
  return(L)
```

```
}
# Download and unzip the image series

file_list = download_unzip_series(
  series_instance_uid = series_instance_uid)
```

```
Downloading Series
```

```
Unzipping Series
```

Here we extracted a single series of a CT brain scan. The data are in DICOM format.

Converting DICOM to NIfTI

We will use `dcm2niix` to convert the data from DICOM to NIfTI. The function `dcm2niix` is wrapped in the `dcm2niir` R package (Muschelli 2018). We will use `dcm2niir::dcm2nii` to convert the file. We use `check_dcm2nii` to grab the relevant output files:

```
library(dcm2niir)
dcm_result = dcm2nii(file_list$dirs)
```

```
#Copying Files
```

```
# Converting to nii
```

```
'/Library/Frameworks/R.framework/Versions/3.6/Resources/library/dcm2niir/dcm2niix' -9 -z y -f %p_%t_%s
```

```
dcm_result$nii_after
```

```
[1] "/var/folders/1s/wrtqcpxn685_zk570bnx9_rr0000gr/T//RtmpJX520z/filed38f203a0be5/HEAD_STD_2001012416
[2] "/var/folders/1s/wrtqcpxn685_zk570bnx9_rr0000gr/T//RtmpJX520z/filed38f203a0be5/HEAD_STD_2001012416
```

```
result = check_dcm2nii(dcm_result)
result
```

```
[1] "/var/folders/1s/wrtqcpxn685_zk570bnx9_rr0000gr/T//RtmpJX520z/filed38f203a0be5/HEAD_STD_2001012416
attr(,"json_file")
[1] "/var/folders/1s/wrtqcpxn685_zk570bnx9_rr0000gr/T//RtmpJX520z/filed38f203a0be5/HEAD_STD_2001012416
```

Here we see the output is a single NIfTI file. If there is any gantry tilt or variable slice thickness, `dcm2niix` has accounted for this. We also see an associated `json` file, which is a BIDS sidecar file.

We can show the `json` file by using `jsonlite::fromJSON`:

```
json = jsonlite::fromJSON(attr(result, "json_file"))
names(json)
```

```
 [1] "Modality"                    "Manufacturer"
 [3] "ManufacturersModelName"      "PatientPosition"
 [5] "ProcedureStepDescription"    "SoftwareVersions"
 [7] "SeriesDescription"           "ProtocolName"
 [9] "ScanOptions"                 "ImageType"
[11] "SeriesNumber"                "AcquisitionTime"
[13] "AcquisitionNumber"           "XRayExposure"
[15] "ReconMatrixPE"               "ImageOrientationPatientDICOM"
[17] "ConversionSoftware"          "ConversionSoftwareVersion"
```

where we see a lot of the information necessary for reporting are given.

Next we read the data into `R` into a `nifti` object:

```r
library(neurobase)
img = readnii(result)
ortho2(img)
```

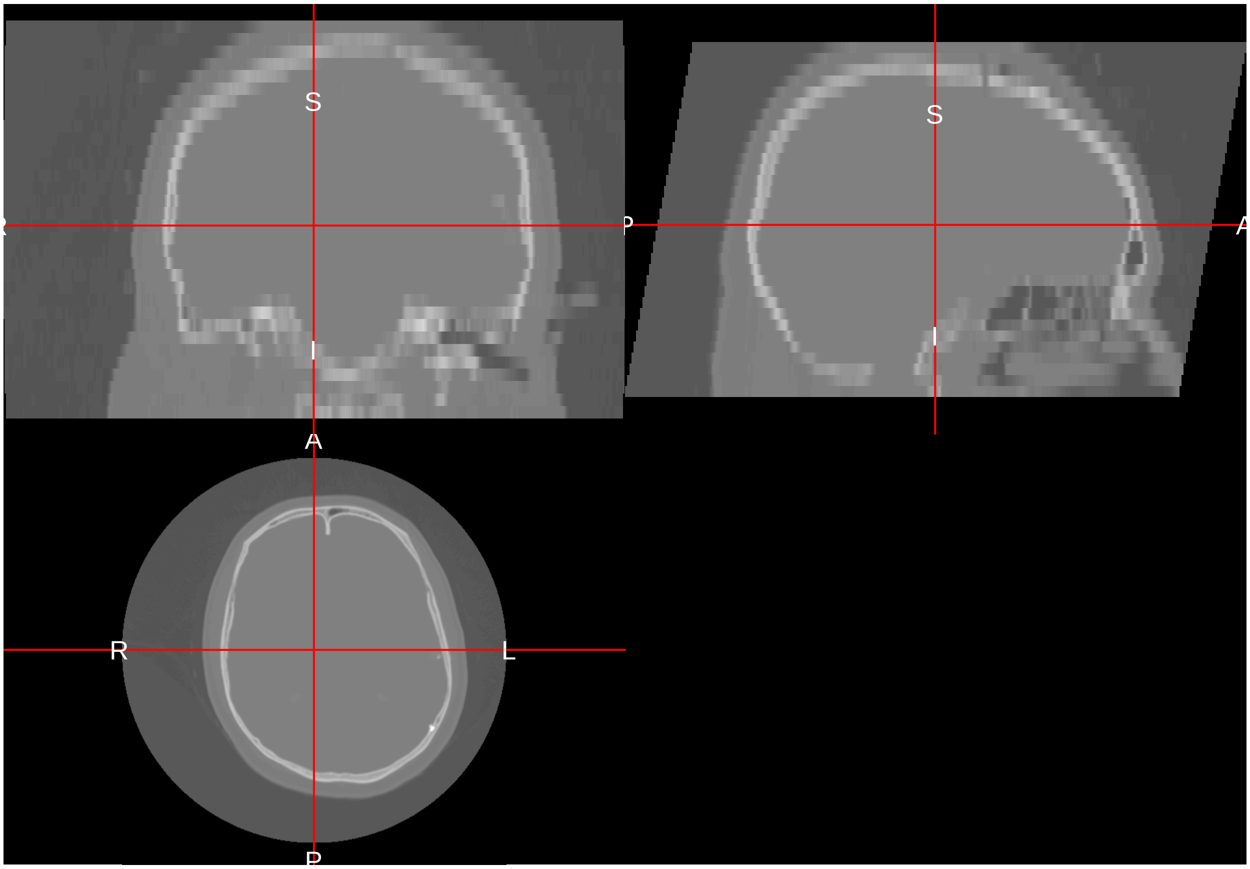


```r
range(img)
```

```
[1] -3024  3071
```

Here we will use `neurobase::rescale_img` to make sure the minimum is -1024 and the maximum is 3071. The minimum can be lower for areas outside the field of view (FOV). Here we plot the image and the Winsorized version to see the brain tissue:

```r
img = rescale_img(img, min.val = -1024, max.val = 3071)
ortho2(img)
```

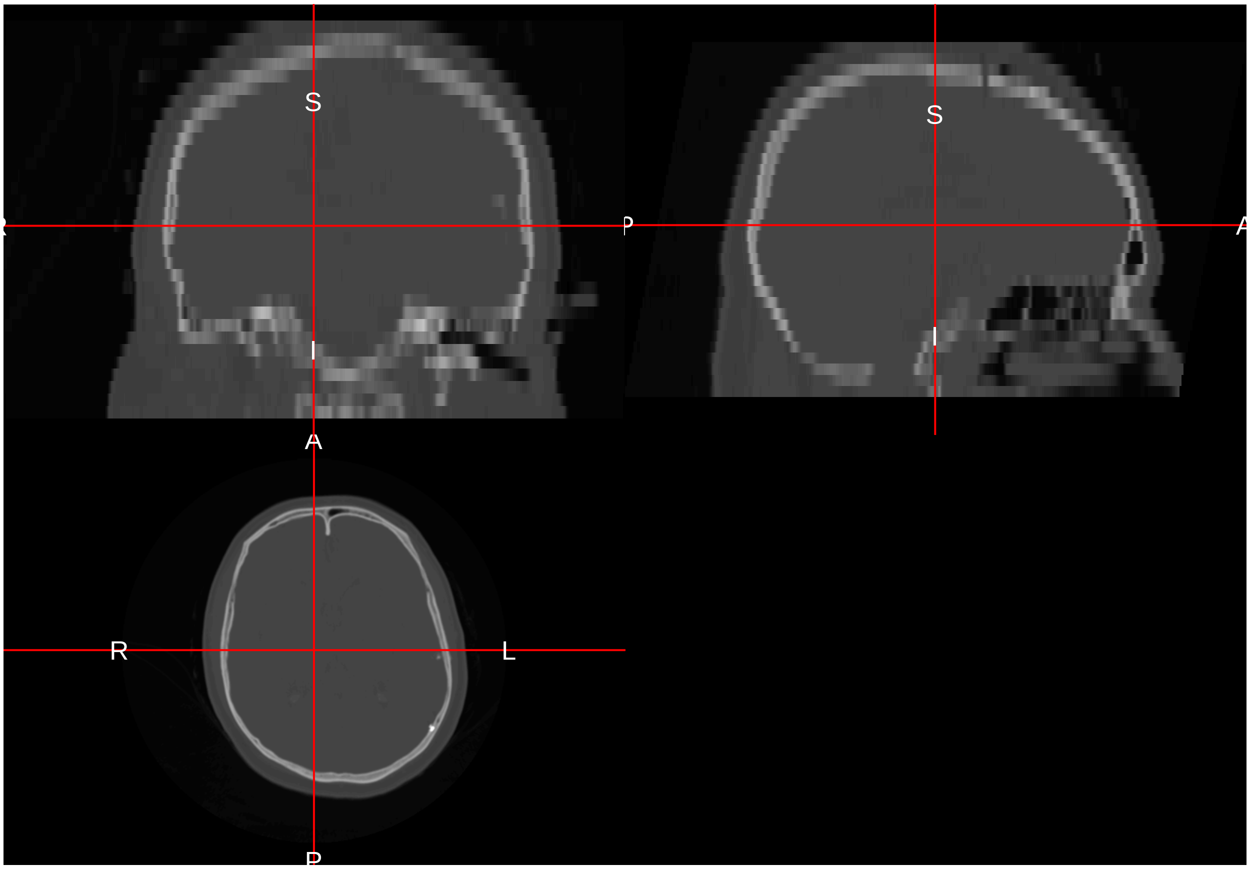


```
ortho2(img, window = c(0, 100))
```

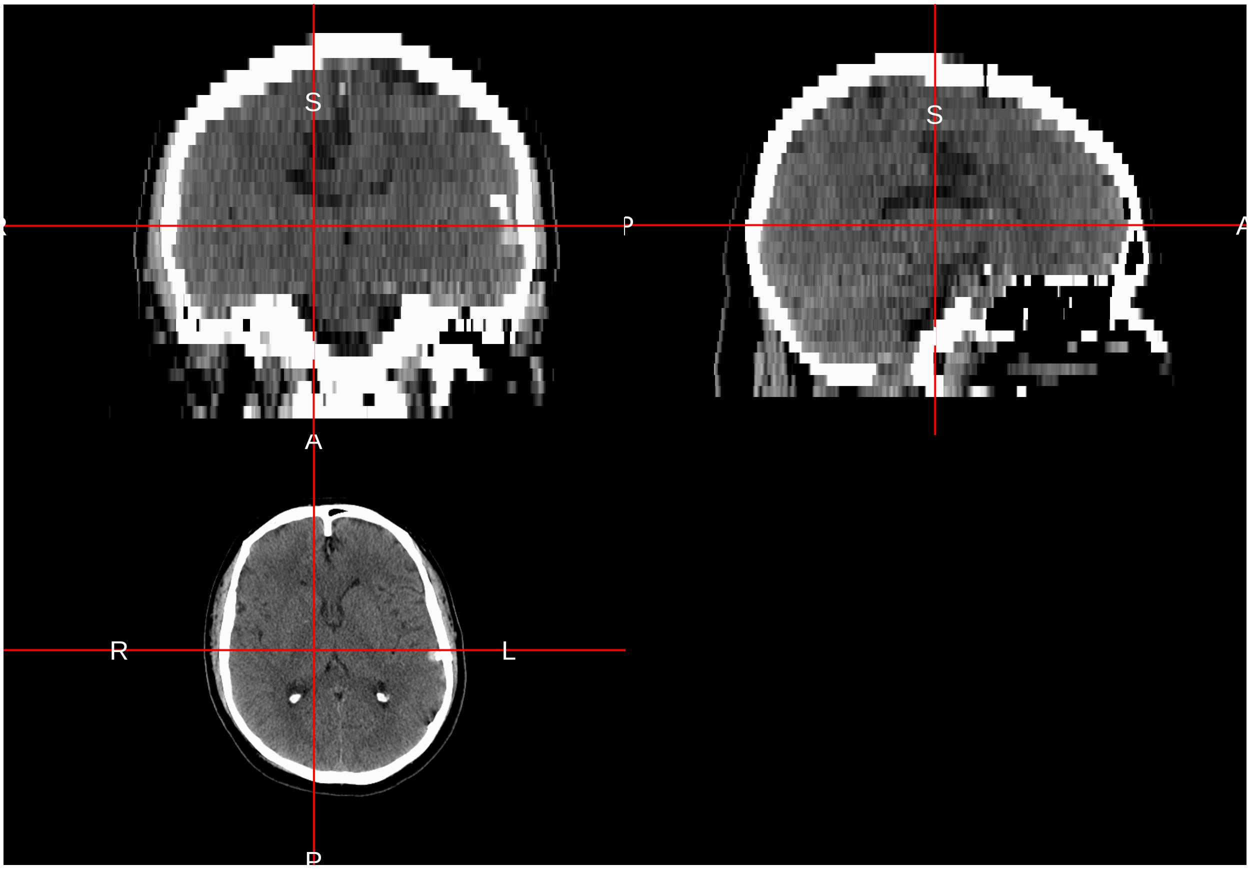

We see the image has high resolution within the axial plane, but not as high resolution in the sagittal plane. We see high values in the skull and other dense areas and lower values within the brain and the darkest values outside of the head.

Skull Strip

We can skull strip the image using `CT_Skull_Strip` or `CT_Skull_Stripper` from the `ichseg` R package. The `CT_Skull_Stripper` has a simple switch to use `CT_Skull_Strip` or `CT_Skull_Strip_robust` (Muschelli 2019b).

```
library(ichseg)
ss = CT_Skull_Strip(img, verbose = FALSE)
```

```
Warning in get.fsl(): Setting fsl.path to /usr/local/fsl
```

```
Warning in get.fsloutput(): Can't find FSLOUTPUTTYPE, setting to NIFTI_GZ
```

```
ortho2(img, ss > 0,
       window = c(0, 100),
       col.y = scales::alpha("red", 0.5))
```

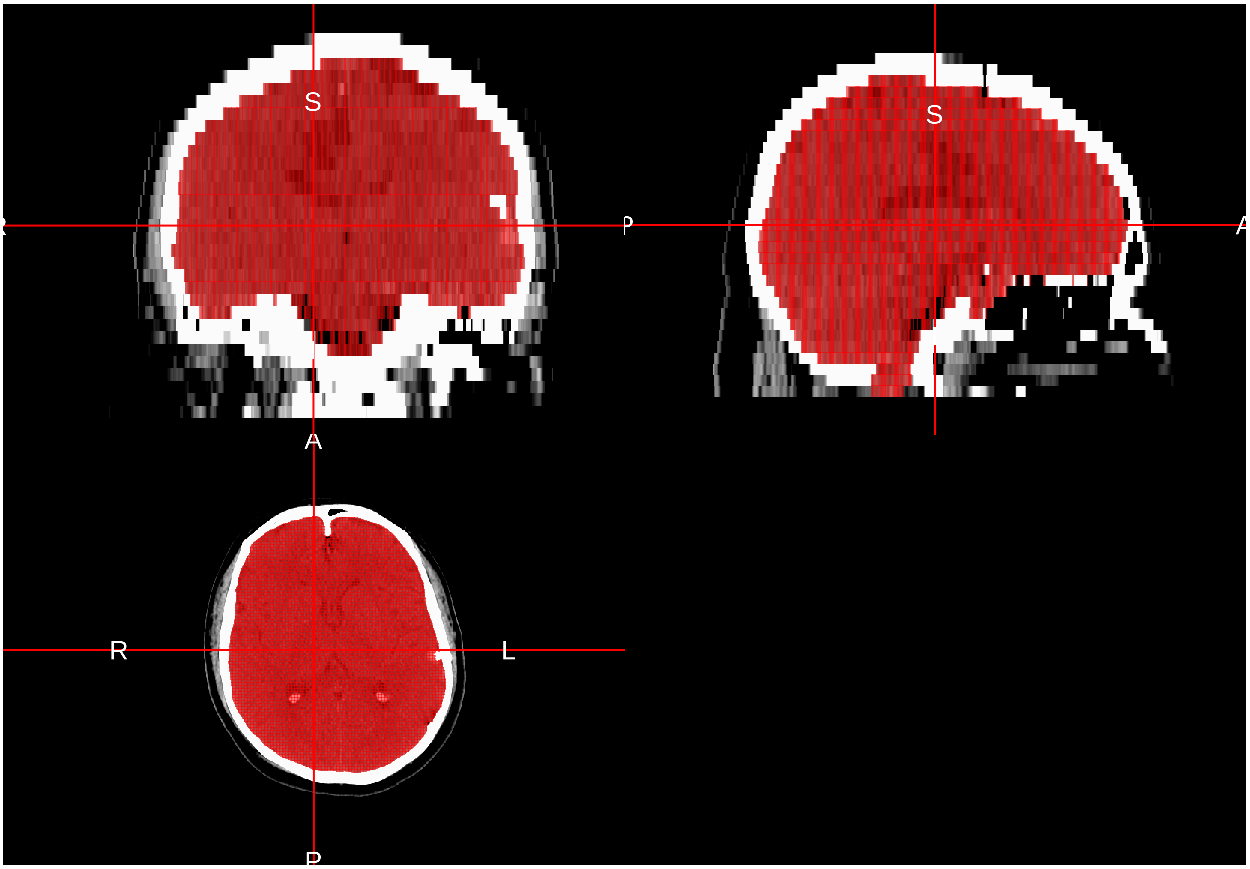

The CT_Skull_Strip_robust function does 2 neck removals using remove_neck from extrantsr and then find the center of gravity (COG) twice to make sure the segmentation focuses on the head, which uses some FSL (Jenkinson et al. 2012) functions in the fslr package (Muschelli et al. 2015). In some instances, the whole neck is included in the scan, such as some of the head-neck studies in TCIA.

(Optional) Defacing the Image

If you have FSL installed, in the fslr R package version (>= 2.23.0), the deface_image function should allow for defacing of the image (Jenkinson et al. 2012; Muschelli et al. 2015). The defacing can be a part of a de-identification protocol to aim for HIPAA compliance:

```
noface_file = fslr::deface_image(img + 1024, template = NULL, face_mask = NULL)
```

```
FSLDIR='/usr/local/fsl'; PATH=${FSLDIR}/bin:${PATH};export PATH FSLDIR; sh "${FSLDIR}/etc/fslconf/fsl.s

FSLDIR='/usr/local/fsl'; PATH=${FSLDIR}/bin:${PATH};export PATH FSLDIR; sh "${FSLDIR}/etc/fslconf/fsl.s

FSLDIR='/usr/local/fsl'; PATH=${FSLDIR}/bin:${PATH};export PATH FSLDIR; sh "${FSLDIR}/etc/fslconf/fsl.s
```

```
noface = readnii(noface_file)
noface = noface - 1024
ortho2(noface)
```

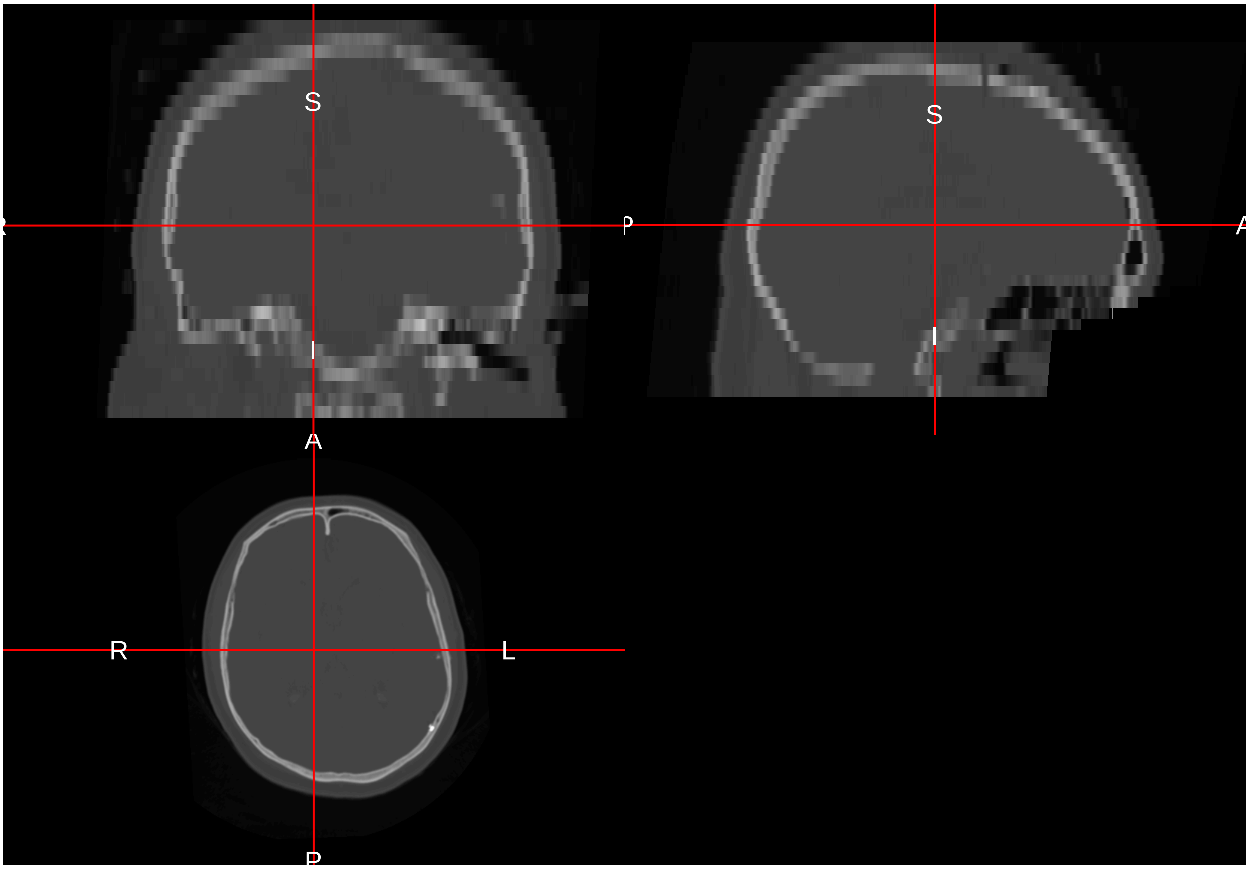

We see that the face has been removed from the image. If you want the mask instead of the image with the face removed, you can run `fslr::face_removal_mask`. Alternatively, the `ichseg::ct_biometric_mask` function should try to get masks of the face and ears, running the `ichseg::ct_face_mask` and `ichseg::ct_ear_mask` functions and combining them into one mask. You can then either remove those areas, randomize the voxels (not recommended), or put a heavy smoother over the area.

Defacing using Quickshear method

If you have the `fslr` package 2.23.1 or above, the `quickshear` method (Schimke and Hale 2011) has been implemented. This is different from the `deface_image` above, but requires a brain mask.

```
qs_noface = fslr::quickshear_deface_image(file = img + 1024, brain_mask = ss > 0)
```

```
Reorienting image to RPI

Getting 2D Mask

Eroding to get outline

Getting Convex Hull

Reorienting image to original orientation
```

```
ortho2(qs_noface - 1024)
```

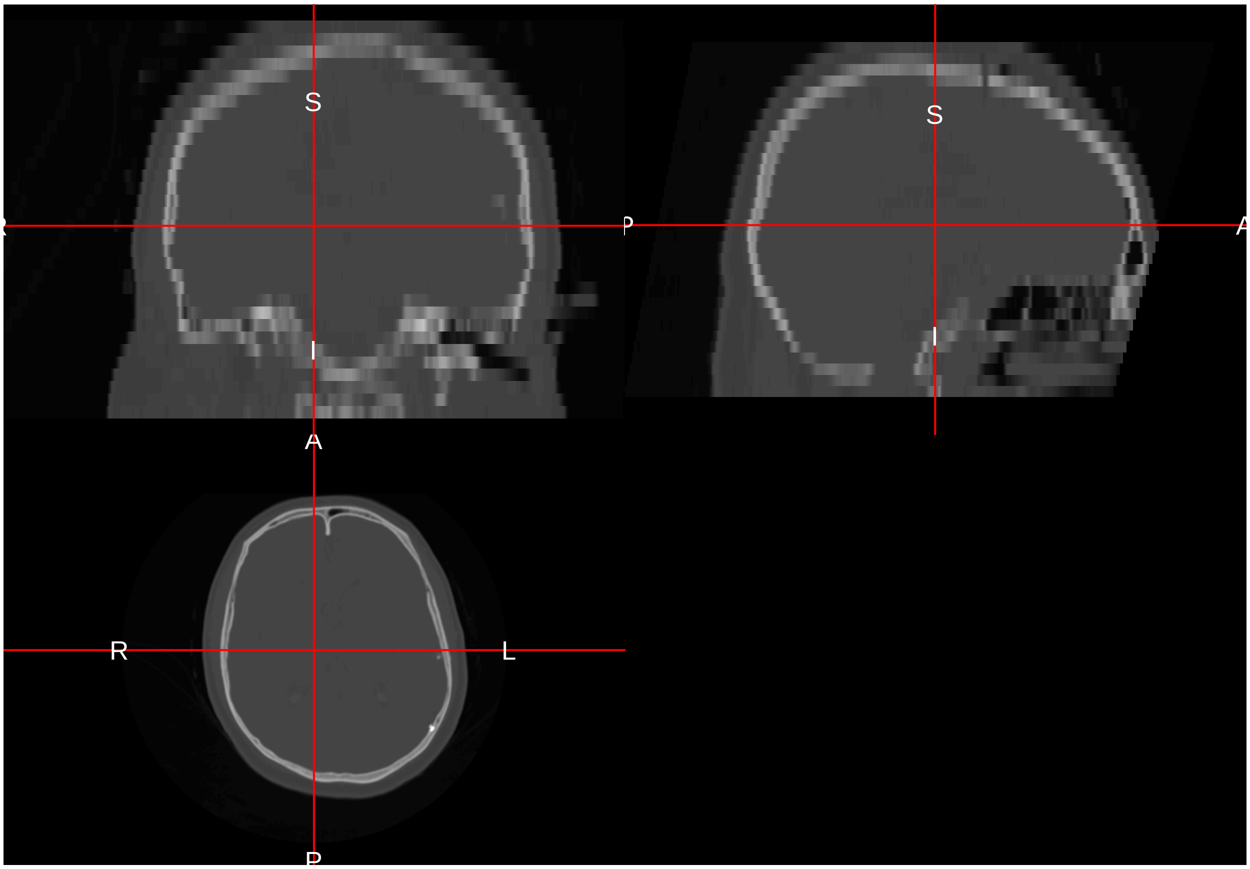

Registration

Here we register the image to the template image from Rorden (2012). We will use the `registration` function from the `extrantsr` R package (Muschelli 2019a). The `extrantsr` package uses the `ANTsR` R package to perform the registration, and simply wraps multiple commands together (Avants 2019). We will use a Symmetric Normalization (SyN) type of registration, which first uses an affine registration, then combines it with a symmetric non-linear diffeomorphism. The output file `reg$outfile` is the registered image.

```
template_image = ichseg::ct_template(type = "image")
ortho2(template_image, window = c(0, 100))
```

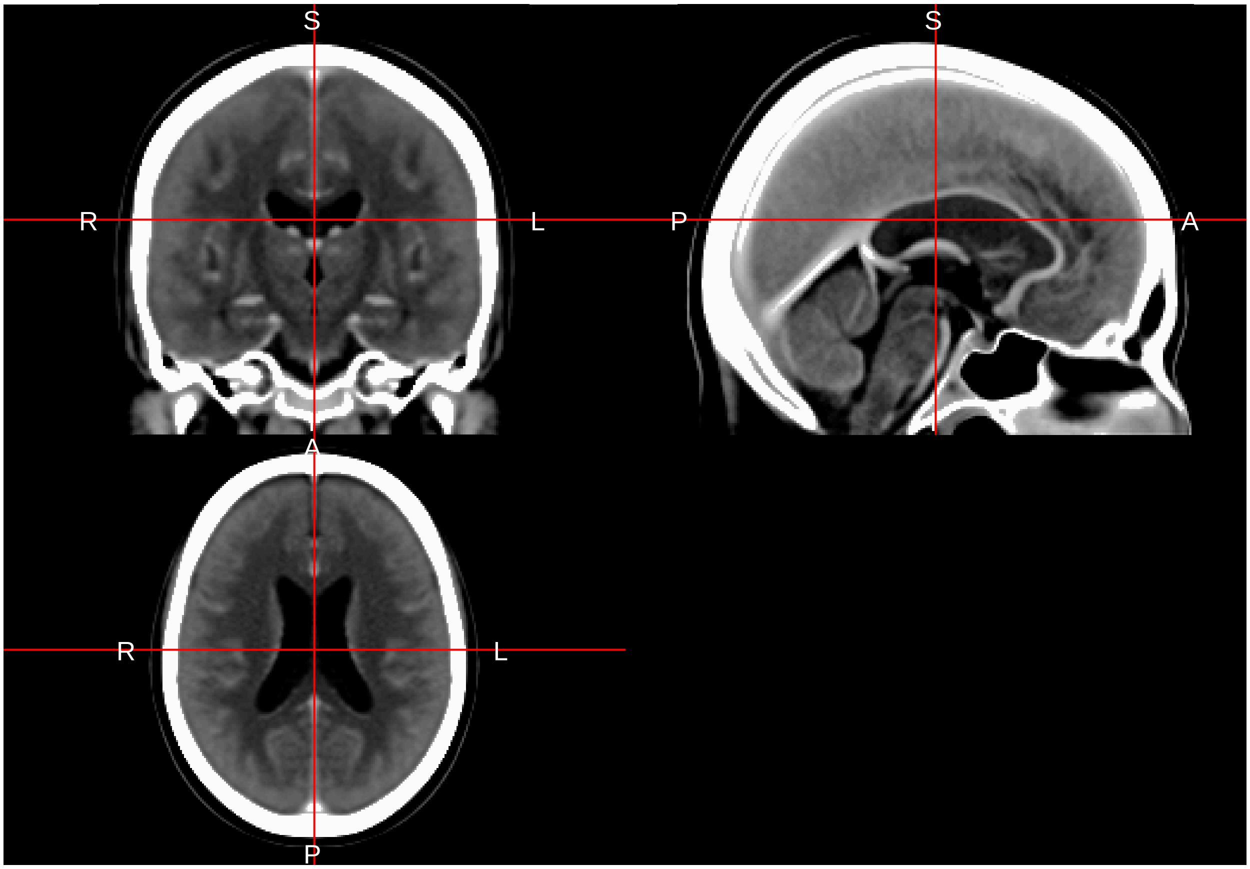


```r
reg = extrantsr::registration(
  img, template.file = template_image,
  typeofTransform = "SyN",
  interpolator = "Linear")
```

```
# Running Registration of file to template

# Applying Registration output is

$fwdtransforms
[1] "/var/folders/1s/wrtqcpxn685_zk570bnx9_rr0000gr/T//RtmpJX520z/filed38f7d8f91631Warp.nii.gz"
[2] "/var/folders/1s/wrtqcpxn685_zk570bnx9_rr0000gr/T//RtmpJX520z/filed38f7d8f91630GenericAffine.mat"

$invtransforms
[1] "/var/folders/1s/wrtqcpxn685_zk570bnx9_rr0000gr/T//RtmpJX520z/filed38f7d8f91630GenericAffine.mat"
[2] "/var/folders/1s/wrtqcpxn685_zk570bnx9_rr0000gr/T//RtmpJX520z/filed38f7d8f91631InverseWarp.nii.gz"

$prev_transforms
character(0)

# Applying Transformations to file

 [1] "-d"
 [2] "3"
 [3] "-i"
 [4] "<pointer: 0x7fb2d63f0b80>"
 [5] "-o"
 [6] "<pointer: 0x7fb2d6314c40>"
```

```
 [7] "-r"
 [8] "<pointer: 0x7fb2ca78dd10>"
 [9] "-n"
[10] "linear"
[11] "-t"
[12] "/var/folders/1s/wrtqcpxn685_zk570bnx9_rr0000gr/T//RtmpJX520z/filed38f7d8f91631Warp.nii.gz"
[13] "-t"
[14] "/var/folders/1s/wrtqcpxn685_zk570bnx9_rr0000gr/T//RtmpJX520z/filed38f7d8f91630GenericAffine.mat"
```

```
# Writing out file
```

```
[1] "/var/folders/1s/wrtqcpxn685_zk570bnx9_rr0000gr/T//RtmpJX520z/filed38f5ac21b51.nii.gz"
```

```
# Reading data back into R
```

```
wimg = window_img(reg$outfile, window = c(0, 100))
double_ortho(template_image, wimg, window = c(0, 100))
```

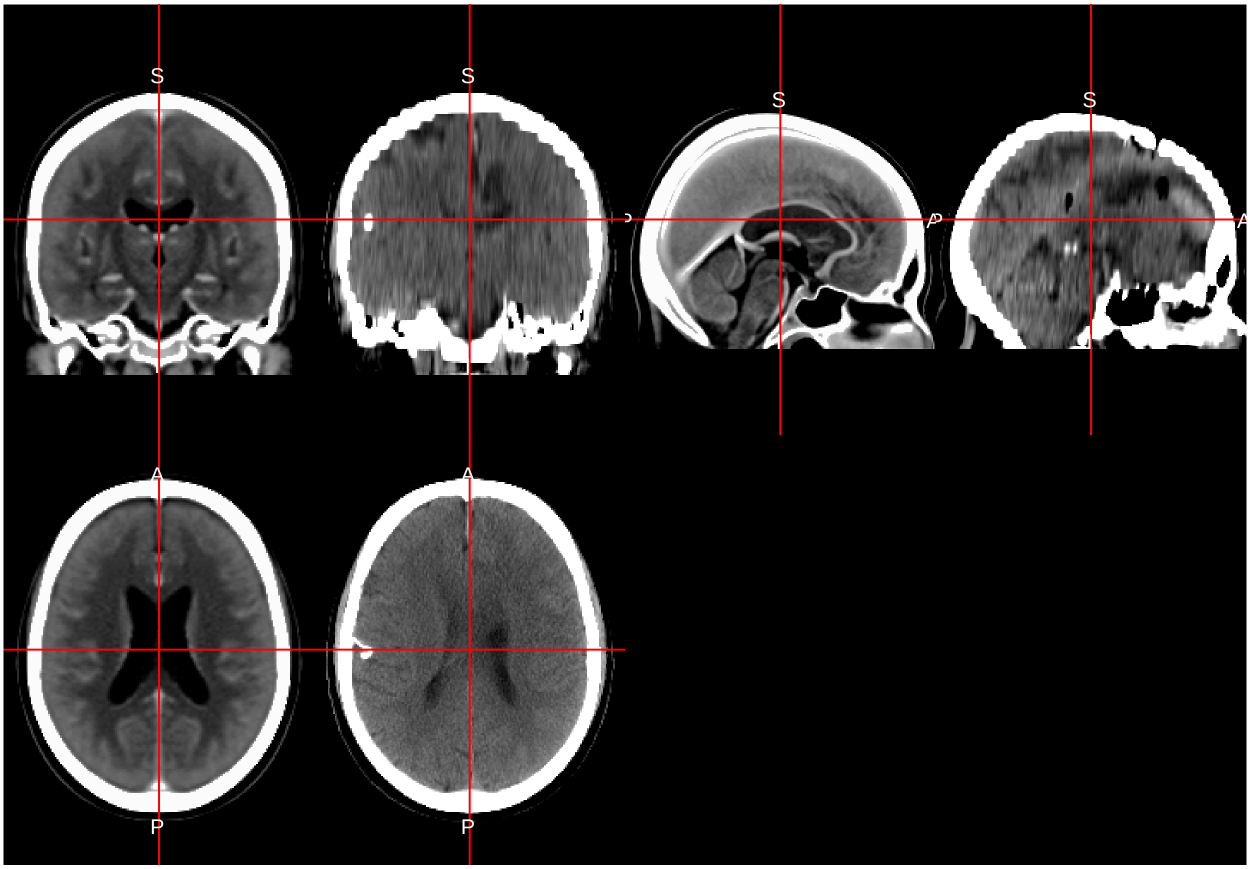

We see relatively good alignment between the template image (left) and the registered image (right)

Here we will use the skull-stripped template and perform the same registration with the skull-stripped image.

```
template_brain = ichseg::ct_template(type = "brain")
ortho2(template_brain, window = c(0, 100))
```

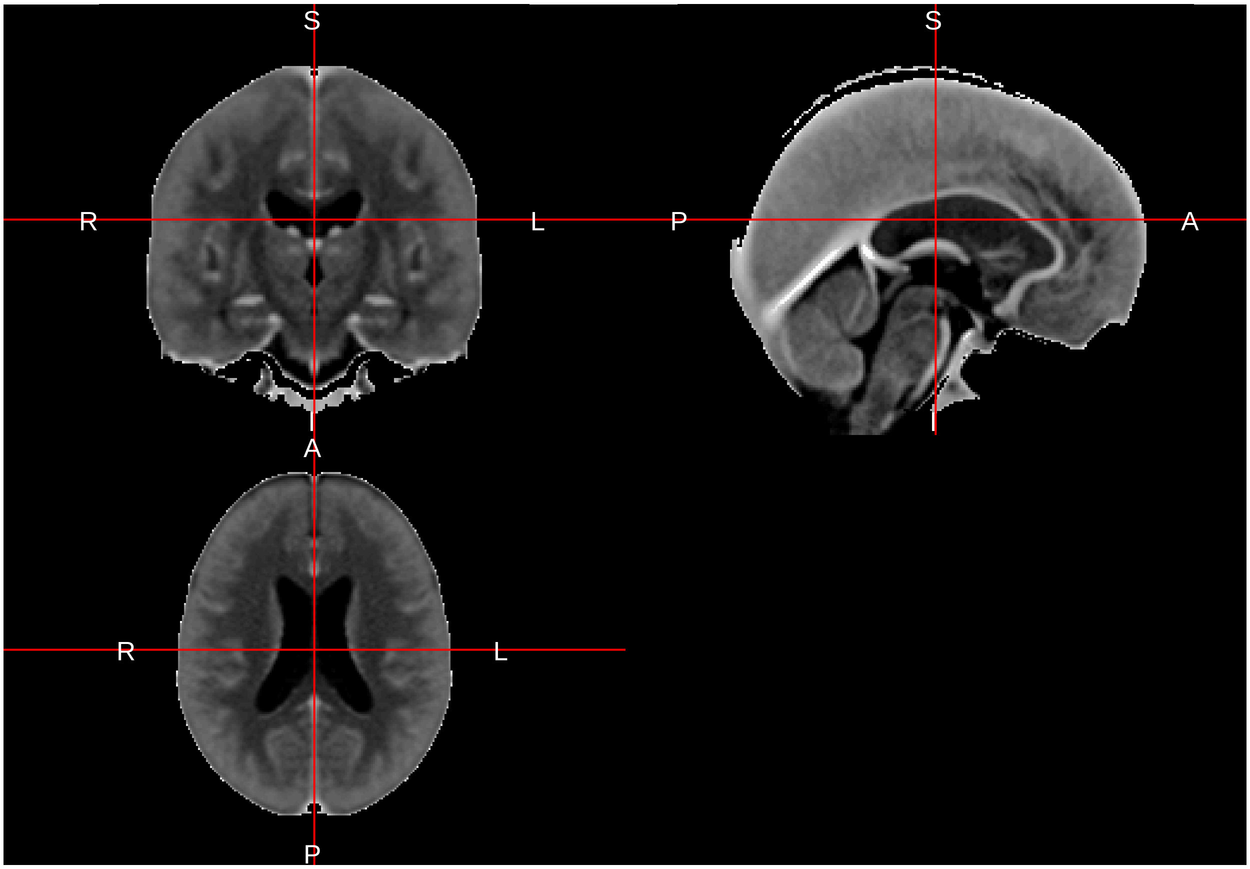


```r
brain_reg = extrantsr::registration(
  ss, template.file = template_brain,
  typeofTransform = "SyN",
  interpolator = "Linear")
```

```
# Running Registration of file to template

# Applying Registration output is

$fwdtransforms
[1] "/var/folders/1s/wrtqcpxn685_zk570bnx9_rr0000gr/T//RtmpJX520z/filed38f455add901Warp.nii.gz"
[2] "/var/folders/1s/wrtqcpxn685_zk570bnx9_rr0000gr/T//RtmpJX520z/filed38f455add900GenericAffine.mat"

$invtransforms
[1] "/var/folders/1s/wrtqcpxn685_zk570bnx9_rr0000gr/T//RtmpJX520z/filed38f455add900GenericAffine.mat"
[2] "/var/folders/1s/wrtqcpxn685_zk570bnx9_rr0000gr/T//RtmpJX520z/filed38f455add901InverseWarp.nii.gz"

$prev_transforms
character(0)

# Applying Transformations to file

 [1] "-d"
 [2] "3"
 [3] "-i"
 [4] "<pointer: 0x7fb2d62559c0>"
 [5] "-o"
 [6] "<pointer: 0x7fb2d6311370>"
```

```
 [7] "-r"
 [8] "<pointer: 0x7fb2d631def0>"
 [9] "-n"
[10] "linear"
[11] "-t"
[12] "/var/folders/1s/wrtqcpxn685_zk570bnx9_rr0000gr/T//RtmpJX520z/filed38f455add901Warp.nii.gz"
[13] "-t"
[14] "/var/folders/1s/wrtqcpxn685_zk570bnx9_rr0000gr/T//RtmpJX520z/filed38f455add900GenericAffine.mat"
```

```
# Writing out file
```

```
[1] "/var/folders/1s/wrtqcpxn685_zk570bnx9_rr0000gr/T//RtmpJX520z/filed38f429ec413.nii.gz"
```

```
# Reading data back into R
```

```r
wbrain = window_img(brain_reg$outfile, window = c(0, 100))
double_ortho(template_image, wbrain, window = c(0, 100))
```

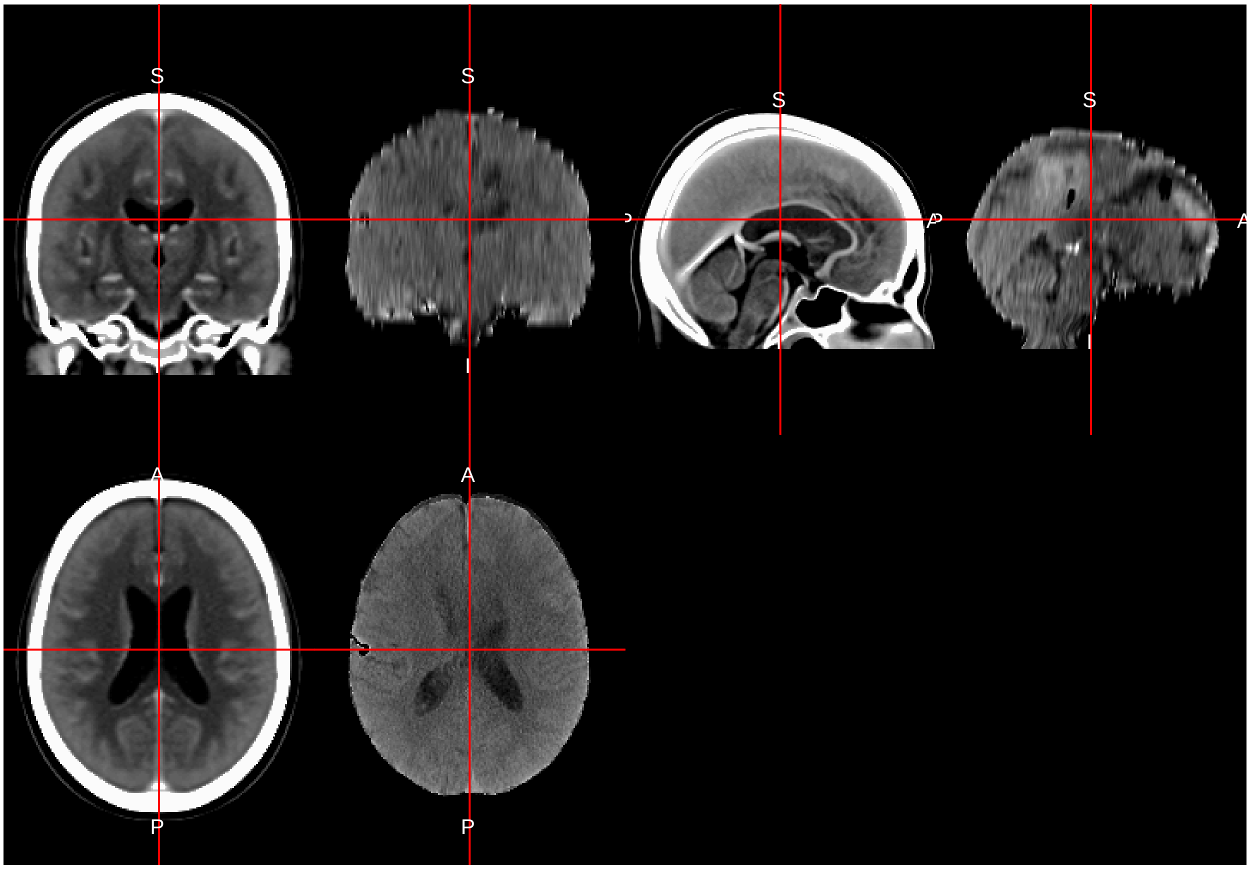

We see again good alignment, but we see that there are some stark differences in these registrations when we compare them:

```r
double_ortho(wimg, wbrain)
```

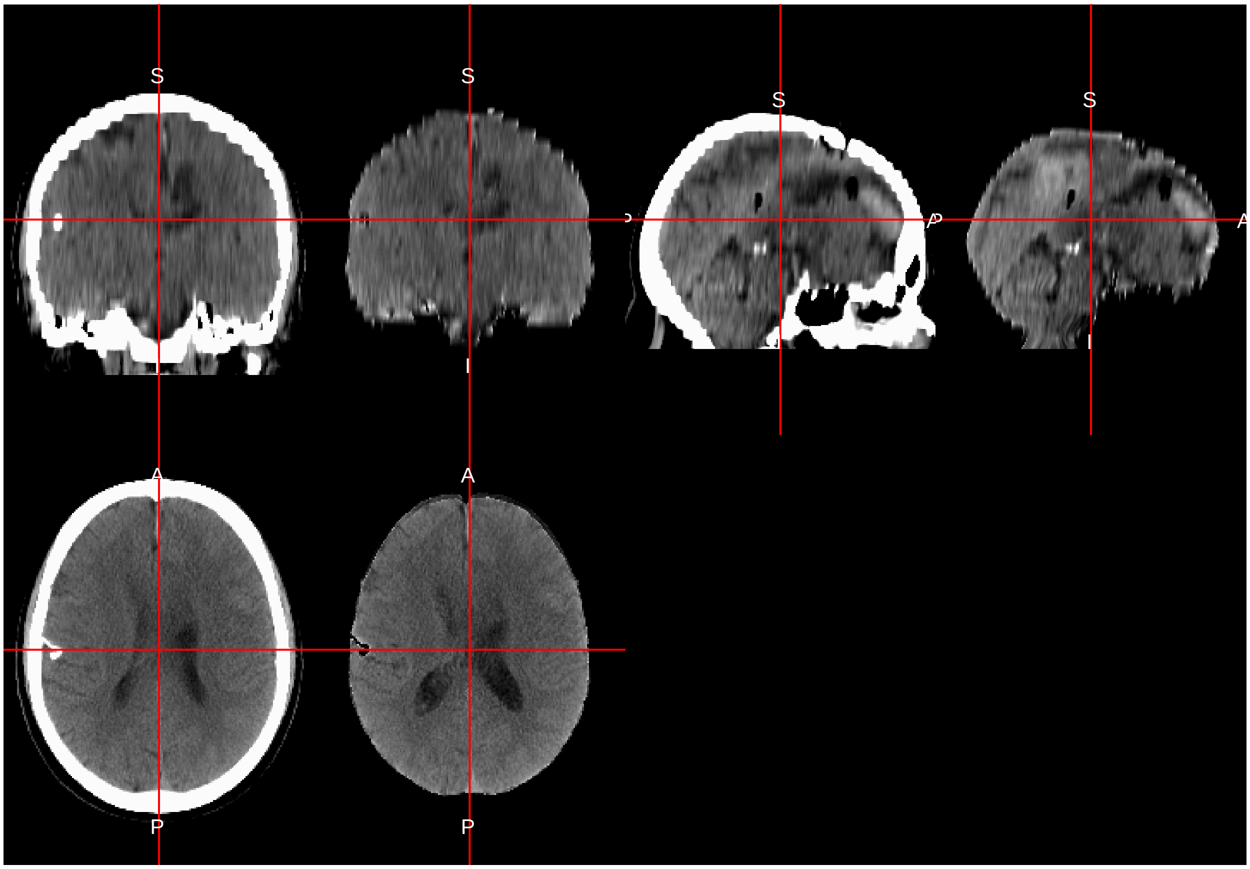

Avants, Brian B. 2019. *ANTsR: ANTs in R: Quantification Tools for Biomedical Images.*

Jenkinson, Mark, Christian F Beckmann, Timothy EJ Behrens, Mark W Woolrich, and Stephen M Smith. 2012. "FSL." *NeuroImage* 62 (2): 782–90.

Muschelli, John. 2018. *dcm2niir: Conversion of DICOM to NIfTI Imaging Files Through R.* https://www.nitrc.org/plugins/mwiki/index.php/dcm2nii:MainPage.

———. 2019a. *extrantsr: Extra Functions to Build on the ANTsR Package.*

———. 2019b. *ichseg: Intracerebral Hemorrhage Segmentation of X-Ray Computed Tomography (CT) Images.*

Muschelli, John, Elizabeth Sweeney, Martin Lindquist, and Ciprian Crainiceanu. 2015. "fslr: Connecting the FSL Software with R." *The R Journal* 7 (1): 163–75.

Russell, Pamela, Kelly Fountain, Dulcy Wolverton, and Debashis Ghosh. 2018. "TCIApathfinder: An R Client for the Cancer Imaging Archive REST API." *Cancer Research* 78 (15): 4424–6.

Schimke, Nakeisha, and John Hale. 2011. "Quickshear Defacing for Neuroimages." In *HealthSec*.
